# Paula Radcliffe: elite athlete and clean air advocate

**DOI:** 10.2471/BLT.19.030319

**Published:** 2019-03-01

## Abstract

Paula Radcliffe tells Gary Humphreys about her journey from athlete to clean air advocate and her hopes for a clean air initiative launched by the International Association of Athletics Federation.

Q: When did you start running?

A: When I was seven years old I used to run with my father who was a keen runner himself. In fact, he ran the London Marathon several times. I only started to get serious about running when I was nine and I joined Frodsham Athletics Club in Cheshire. Then I saw Ingrid Kristiansen break the world marathon record at the London Marathon in 1985, and that really inspired me. The following year my parents moved to Bedfordshire and I joined the Bedford & County Athletics Club.

Q: Was that when you started competing?

A: No, I was already competing for the Frodsham Harriers, but it was a while before I won at the national level. I competed in my first race at the national level – the girls English Schools Cross Country Championships – when I was 12, and I came in 299th out of a field of 600. The following year I came in fourth, and a couple of years later I won the English Schools 1500 metres title. That was my first big win.

Q: Had you already been diagnosed with asthma at that point?

A: No, I was diagnosed with asthma when I was about 14. I was getting dizzy after training sessions and having trouble breathing. My mum took me to the doctor after I blacked out in training.

Q: Do you know what triggered it?

A: I think it was a culmination of things. We had moved from Cheshire down to Bedfordshire the year before and there was a lot more oilseed rape (*Brassica napus*) in the fields, which it turned out was one of my main triggers. Also, it was more of a built-up area and so there was a bit more pollution.

Q: You must have wondered if your running days were over.

A: Nothing was ever going to stop me running, but I was worried that the doctors were going to say you’re not going to be able to run anymore. Luckily, my family doctor was very forward-thinking. He said you’re going to have to use inhalers and learn how to manage and control your condition, but he also said that doing sport is going to make your lungs stronger, which was exactly what I wanted to hear. He also gave me a peak flow monitor, which is a tube that measures the volume of air that you can blow out fast and hard in one breath. The idea was that I would be able to monitor my asthma and see when I needed to increase the dosage of my asthma preventer inhaler.

“Athletes are not just exposed to polluted air, they actually monitor their exposure.”

Q: So you were in control at that point.

A: Exactly. It made a huge difference. It took away a lot of the uncertainty and the anxiety that went with it and allowed me to compete and train as hard as I wanted to. It also made me a lot more aware of the air I was breathing. Later, my approach to air-quality monitoring became more sophisticated. I had sports science teams looking at which specific types of pollen would trigger my asthma so that I could avoid certain training areas. But I still used my basic peak flow monitor, and still do.

Q: One way or another you’ve been preoccupied with air quality for a long time.

A: I certainly have, especially as a marathon runner. You have to understand that a marathon runner breathes in as much air in three hours as a sedentary person would over two days, and the air he or she is breathing is not always the cleanest. So someone like me or Haile Gebreselassie, who is also a clean air ambassador for the International Association of Athletics Federation (IAAF), and also has asthma, is going to be very exposed to air pollution.

Q: Didn’t Haile Gebreselassie withdraw from the marathon at the 2008 Beijing Olympics because of the air quality there?

A: He did. He was afraid that breathing the air would harm his chances of breaking the marathon world record, which was something he wanted to do. So he ran in the stadium for the 10 000 metres, but he didn’t run in the streets.

Q: How did you get involved in clean air advocacy?

A: I was approached by the IAAF to be an ambassador for their clean air initiative and I accepted. I was already working in advocacy, having been with Asthma UK since 1998, a charity I continue to work with because it means a lot to me at a personal level.

Q: What is the focus of your advocacy work with Asthma UK?

A: Getting across the message that youngsters who are diagnosed with asthma can do whatever they set their minds to. I tell them, you should control your asthma and not let it control you. That means taking the right medication and learning to manage their condition.

Q: Can you tell me a little about the clean air initiative?

A: It’s a joint effort of the United Nations Environment Programme (UNEP) and the IAAF which has started a programme to monitor the air in athletics stadiums around the world. The partnership is going to be supported by the Climate and Clean Air Coalition (CCAC) which is going to create an air quality monitoring network.

Q: Why the focus on stadiums?

A: Because stadiums are where the athletes are. Elite athletes have a key role to play in raising awareness about the health impacts of air pollution. Not just because they have the visibility, but because they are particularly exposed to the problem they’re trying to help address. Also, athletes are not just exposed to polluted air, they actually monitor their exposure. Monitoring performance and air quality over time is going to give us a much better understanding of the impact of poor air quality on our bodies. So we want to use our visibility, our exposure and monitoring know-how to bring this issue to the fore.

Q: Has the monitoring of stadiums begun?

A: Yes. Monitoring devices have already been installed in four stadiums, the most recent being at the Mexican Olympic Sports Center in Mexico City. The others have been installed in Monaco, Ethiopia and Australia. The plan is to install up to 1000 monitors over the next four years, spread across stadiums in the 214 countries of the federation.

“People are being encouraged to run for their health, but if the air they are running in is polluted they are actually being exposed to health risks.”

Q: What exactly are the monitors designed to track?

A: A variety of pollutants, including fine particulate matter down to 2.5 microns which, as you know, not only lodges deep inside the lungs, but can cross the lung barrier and enter the blood stream. Chronic exposure to such particles is known to contribute to the risk of developing cardiovascular and respiratory diseases, as well as lung cancer. The monitors will also track levels of ozone and nitrogen dioxide.

Q: What will the IAAF do with the data once they have it?

A: They’ll use it to establish a basis for education and awareness-raising, and to inform discussions about the air quality issue and policy development. The IAAF Health and Science Department also hopes to be able to study the correlation between air quality and athletes’ performance. Finally, data will be used to help runners choose the best times of day to run in their cities, as a kind of public service.

Q: To reduce runners’ exposure to pollution?

A: Absolutely. It’s estimated that about a half billion people run on a regular basis, and many of those people are running in cities where the air quality is poor. According to WHO surveys, only about 20% of the world’s urban population lives in areas that comply with air quality standards considered safe by WHO. People are being encouraged to run for their health, but if the air they are running in is polluted, they are actually being exposed to health risks. This is especially true in cities. If you go for a run in a city like New Delhi in India, as I did a few months ago, only the first five minutes is beneficial for your health because you are exercising; beyond that point the pollution that you are inhaling is harming you. We need to give runners information so they have choices about where it is good to run and what time of day is good to run. Ultimately, of course, we need to encourage cities to clean up their air.

Q: What do you hope the clean air initiative will achieve?

A: An estimated seven million people a year die from illnesses related to air pollution. We need to act now to address that, starting by getting a better understanding of the impact of poor air quality on our bodies. But we also need to make people more aware of the problem, so they can put pressure on their governments to introduce the policies needed to bring about change. My hope is that the clean air initiative will help achieve those goals.

